# Evaluation of Effect of *Taxus baccata* Leaves Extract on Bronchoconstriction and Bronchial Hyperreactivity in Experimental Animals

**DOI:** 10.4103/0975-1483.76418

**Published:** 2011

**Authors:** PK Patel, KV Patel, TR Gandhi

**Affiliations:** *Department of Pharmacology, C. K. Pithawalla Institute of Pharmaceutical Science and Research, Surat - 395 007, Gujarat, India*; 1 *Department of Pharmacology, Anand Pharmacy College, Anand - 388 001, Gujarat, India*

**Keywords:** Anti-asthmatic activity, alcoholic extract, leaves, *Taxus baccata*

## Abstract

The present investigation was undertaken to evaluate the bronchodilating effect and bronchial hyperreactivity of alcoholic extract of *Taxus baccata* Linn. (AET) leaves in experimental animals. Bronchodilator activity of AET was studied on the histamine and acetylcholine aerosol induced bronchospasm in guinea pigs and bronchial hyperreactivity was studied on bronchoalveolar lavage fluid (BALF) in the egg albumin sensitized guinea pigs and by histopathological studies. *In vitro* mast cell stabilizing activity was studied using compound 48/80 as a degranulating agent. Treatment with AET (200 and 400 mg/kg, p.o., for 7 days) showed significant protection against histamine and acetylcholine aerosol induced bronchospasm in guinea pigs. Significant decrease in the total leukocyte and differential leukocyte count in the BALF of the egg albumin sensitized guinea pigs was observed by administration of AET (200 and 400 mg/kg, p.o., for 15 days). AET dose dependently protected the mast cell disruption induced by compound 48/80. These results suggest that AET not only has bronchodilating activity but also decreases bronchial hyperreactivity by decreasing the infiltration of inflammatory cells in the airway and inhibiting the release of histamine like mediators from the mast cell by stabilizing it.

## INTRODUCTION

Asthma is one of the most common disorders encountered in clinical medicine in both children and adults, characterized by inflammation of the airway that is central to airway dysfunction. It is known that asthma can be triggered by various factors: allergens, drugs, respiratory infection, dust, cold air, exercise, emotions, occupational stimuli, chemicals, histamine, etc.[1] Histological examination of bronchial biopsies and cytology of bronchoalveolar lavage fluid (BALF) have demonstrated infiltrating inflammatory cells in the tracheobronchial mucosa and airway lumen of patients with asthma, even those with mild disease.[2,3] The influx of inflammatory cells is accompanied by marked and characteristic pathophysiological changes to the airways, including thickening of the airway wall, which have been implicated in the restriction of airflow and the development of airway hyperresponsiveness.[3] The disease statistics clearly necessitates the increasing need for drugs targeting the mechanisms involved in eosinophil and neutrophil activation and accumulation, for the management of asthma. Glucocorticosteroids are the only drugs currently available that effectively reduce airway inflammation in asthma.[4]

As a result, there is high prevalence of usage of complementary and alternative medicines for treatment of this disease.[5] Ayurveda, an ancient system of Indian medicine, has recommended a number of drugs from indigenous plant sources for the treatment of bronchial asthma and allergic disorders.[6] *Taxus baccata* Linn. (Taxaceae) is an evergreen tree, usually 6 m in height and 1.5–1.8 m in width, found in the temperate Himalayas at an altitude between 1800 and 3300 m and in the hills of Meghalaya and Manipur at an altitude of 1500 m.[7] *T. baccata* has been used in the Ayurvedic system for the treatment of cancer, diarrhea, asthma, hemoptysis and also used as carminative, expectorant, stomachic, etc.[8] *T. baccata* leaves are reported to be used in traditional medicine as abortifacient, antimalarial, antirheumatic and for bronchitis,[9–11] and dried leaves and barks are used against asthma.[12] Anticancer,[13] anti-inflammatory and antinociceptive,[14] antifungal,[15] antimycobacterial[16] activity of *T. baccata* has been reported. Many Ayurvedic practitioners prescribe decoction of leaves of *T. baccata* for the treatment of asthma. However, no scientific studies have been carried out to investigate anti-asthmatic effect in the form of bronchorelaxation and inhibition of bronchial hyperreactivity of leaves of *T. baccata*. In present study, the anti-asthmatic activity of AET was evaluated in experimental animals by using various *in vivo* and *in vitro* models.

## MATERIALS AND METHODS

### Chemicals

Compound 48/80 was purchased from Sigma-Aldrich Chemical Co. (Bangalore, India). Acetylcholine, egg albumin and other chemicals were purchased from S. D. Fine Chem. Ltd. (Mumbai, India) and histamine was purchased from Himedia Laboratories Pvt. Ltd. (Mumbai, India). Ketotifen was obtained as gift sample from Elysium Pharmaceutical Ltd. (Baroda, India). All other chemicals used were of analytical grade.

### Plant material

Dried leaves of *T. baccata* were purchased from a commercial supplier of Mumbai, India. The plant was authenticated by Prof. Minoo Parabia, Head of Department of Bioscience, Veer Narmad South Gujarat University, Gujarat, India, where a plant specimen has been deposited with the no. HMG/0404/2007.

### Preparation of extract

The leaves were reduced to coarse powder and macerated with alcohol (ethanol) for 48 hours, filtered and filtrate was evaporated under reduced pressure to obtained brown crystalline powder. The extract was stored in cool and dry place and used for pharmacological evaluation (alcohol extractive value 4.5% w/w). After obtaining the dry extract, qualitative preliminary phytochemical screening was performed to find out the presence of various phytochemicals.[17] For pharmacological evaluation, the extract was dissolved in distilled water prior to its use.

### Experimental animals

Wistar rats (175–200 g) and guinea pigs (400–600 g) of either sex, housed in standard conditions of temperature (22 ± 2°C), relative humidity (55 ± 5%) and light (12 hours light/dark cycles), were used. They were fed with standard pellet diet and water *ad libitum*. In addition to pellet diet, the guinea pigs were supplemented with Lucerne. The experimental protocol was approved by Institutional Animal Ethical Committee as per the guidance of CPCSEA, Ministry of Social Justice and Empowerment, Government of India (Protocol No. Project 5005). A minimum of six animals were used in each group. Throughout the experiments, the animals were processed according to the suggested ethical guideline for the care of laboratory animals.

### Acute toxicity study

Acute toxicity study was performed on female albino rats according to Organization for Economic Cooperation and Development-425 (OECD-425) guideline. Animals were observed for the next 14 days. There was no sign of changes in behavioral and autonomic profiles and any sign of toxicity or mortality up to a dose of 2000 mg/kg.

### Histamine and acetylcholine aerosol induced bronchospasm in guinea pigs

Experimental bronchial asthma was induced in guinea pigs by exposing them to histamine and acetylcholine aerosol.[18] Guinea pigs were selected and divided into four groups, each containing six animals, out of which groups I and group II were exposed to 0.1% w/v of histamine dihydrochloride aerosol and group III and group IV were exposed to 0.5% w/v of acetylcholine bromide aerosol in histamine chamber (Inco Ltd., Ambala, India). The animals exposed to histamine and acetylcholine aerosol showed progressive dyspnea. The end point preconvulsion dyspnea (PCD) was determined from the time of aerosol exposure to the onset of dyspnea leading to the appearance of convulsion. As soon as the PCD commenced, the animals were removed from chamber and placed in fresh air. This time of PCD was taken as day 0 value. The guinea pigs of group I and group III were treated with the AET 200 mg/kg, p.o. and group II and group IV animals were treated with 400 mg/kg, p.o., once a day for 7 days, after aerosol exposure on day 0. On the 7^th^ day, 2 hours after the last dose, the time for the onset of PCD was recorded as on day 0. The percentage increase in the time of PCD was calculated using following formula:[19]

percentage increase in the time of PCD = $\left(1-\frac{T_{1}}{T_{2}}\right)\times 100$

where T_1_= time for PCD onset on day 0, T_2_= time for PCD onset on day 7.

### Studies on BALF in egg albumin sensitized guinea pigs[20]

Guinea pigs were selected and divided into five groups, i.e., group I (control: distilled water 10 ml/kg); group II (sensitized); group III (Sensitized + prednisolone 5mg/kg, i.p.), group IV (sensitized + *T. baccata* 200 mg/kg, p.o.); and group V (sensitized + *T. baccata* 400 mg/kg, p.o.), each containing six animals. The guinea pigs of group II, group III, group IV and group V were sensitized with egg albumin (1 ml, 10% w/v, i.p.) on the 1^st^ day. The animals of group III were dosed once daily for 15 days with prednisolone 5 mg/kg, while group IV and group V animals were dosed once daily for 15 days with AET. Two hours after the last dose of drug administration (on 15^th^ day), all the animals of group II, group III, group IV and group V were again challenged with egg albumin (0.5 ml, 2% w/v, i.v.) through saphenous vein. After 3 hours of administration of egg albumin or just prior to death of animals, whichever was earlier, the trachea was immediately cannulated after anesthetization and the airways lavaged with saline at 25°C (two aliquots of 1 ml/100 g body weight). Bronchoalveolar cells were collected in two successive lavages using saline and recovered through a tracheal cannula. The BALF was stored on ice and total WBC cell counts were performed using a light microscope. Dilutions of lavage fluid (1 in 10) were made in saline, and differential WBCs were counted by light microscopy stained with Leishman’s stain. At least 200 cells were counted on each slide. Cells were differentiated using standard morphological criteria. All differential cell counts were performed blind and in randomized order at the end of the study. The results obtained were compared between control and sensitized groups and sensitized and treated groups.

### Lung histology

The same animals of the above model, i.e., used for studies on the BALF, were used for the histological study of the lungs. Left bronchi were tied before collection of BALF to avoid possible traumatic damage due to BALF. The lungs were removed and then fixed by slowly inflating with buffer formalin and subsequently embedded in paraffin. A transverse section (2–4 μm thick) was cut from each of the collected lungs and stained with hematoxylin and eosin. Histopathology assessment under light microscope was performed on sections.

### *In vitro* mast cell degranulation by compound 48/80

The effect of *T. baccata* on *in vitro* mast cell degranulation by compound 48/80 was studied following the method of Gupta and Srimal.[21] Normal saline (5 ml/kg) containing 5 units/ml of heparin was injected in the peritoneal cavity of male rats (*n* = 6) lightly anesthetized with ether. After a gentle abdominal massage, the peritoneal fluid containing mast cells was collected in centrifuge tubes placed over ice. Peritoneal fluid of rats was collected and centrifuged at 2000 rpm for 5 min. Supernatant solution was discarded and the cells was washed twice with saline and resuspended in 1 ml of saline. All the solutions were prepared in normal saline.

The peritoneal cell suspension was divided into six parts, viz., –ve control, +ve control, reference standard (ketotifen 10 μg/ml), AET of three concentrations, i.e., 500, 750, 1000 μg/ml, each containing 0.1 ml of cell suspension and incubated at a constant temperature in a water bath at 37°C for 15 min. Then, 0.1 ml of compound 48/80 (10 μg/ml) was added to all the samples except in –ve control and the suspensions were further incubated for 10 min at 37°C. The cells were then stained with 10% of Toluidine blue solution and observed under the high power of light microscope. The percentage granulated and percentage degranulated mast cells were counted. In +ve control group, compound 48/80 was added without the addition of test agents, i.e., ketotifen and *T. baccata*, and in –ve control group neither compound 48/80 nor the test agents were added to correct for spontaneous degranulation of mast cells without any degranulating agent.

### Statistical analysis

The results of various studies were expressed as mean ± SEM and analyzed statistically using Student’s t-test to find out the level of significance. Data were considered statistically significant at minimum level of *P* < 0.05.

## RESULTS

### Acute toxicity study

AET did not produce mortality and any sign of toxicity up to dose of 2000 mg/kg.

### Phytochemical screening

Preliminary qualitative phytochemical screening of AET showed the presence of lignans, flavonoids, glycosides, sugars, amino acids and triterpenoids.

### Effect on histamine and acetylcholine aerosol induced bronchospasm in guinea pigs

AET significantly and dose dependently increased the time of PCD following histamine (*P* < 0.001) and acetylcholine (*P* < 0.01) aerosol induced bronchospasm in guinea pigs [Table 1]. Increase in the time of PCD was more against histamine aerosol as compared to acetylcholine aerosol, following administration of *T. baccata* leaves extract.

**Table 1:** Effect of *Taxus baccata* (p.o., for 7 days) on histamine and acetylcholine aerosol induced bronchospasm in guinea pigs

Groups	Preconvulsion dyspnea time (sec)		
	Before treatment (control)	After treatment	% Increase in the time of PCD
Histamine aerosol (0.1% w/v)			
I- *T. baccata* (200 mg/kg)	125.8 ± 18.56	47.2 ± 25.34*	72.82 ± 3.14
II- *T. baccata* (400 mg/kg)	127.4 ± 20.32	632.7 ± 53.47*	80.67 ± 6.23
Acetylcholine aerosol (0.5% w/v)			
III- *T. baccata* (200 mg/kg)	149.27 ± 11.23	354.5 ± 32.09#	58.73 ± 3.09
IV- *T. baccata* (400 mg/kg)	137.4 ± 23.60	438.57 ± 43.71*	67.98 ± 4.61

Values are expressed as mean ± SEM for six guinea pigs in each group

* *P* < 0.001

# *P* < 0.001 when compared with control group

### Effect on BALF in egg albumin sensitized guinea pigs

After 15 days, the guinea pigs were again challenged with egg albumin. In the BALF, significant increases in the total leukocyte count and differential leukocytes count were observed in the sensitized, i.e., group II (*P* < 0.001) animals as compared to the control, i.e., group I animals. AET (200 and 400 mg/kg, p.o., for 15 days) significantly and dose dependently decreased the total leukocyte count (*P* < 0.05) and differential leukocyte count (*P* < 0.001) in group IV and group V as compared to group II [Table 2] animals. Prednisolone also significantly decreased total leukocytes count (P < 0.001) and differential leukocytes count (*P* < 0.001) compared to sensitized group.

**Table 2:** Effect of *Taxus baccata* (p.o., for 15 days) on BALF in egg albumin sensitized guinea pigs

	Control	Sensitized	Prednisolone (5 mg/kg)	Sensitized + *T. baccata* (200 mg/kg)	Sensitized + *T. baccata* (400 mg/kg)
TLC/mm^3^	8842 ± 429	14,740 ± 670.9*	9247 ± 467.7$	11,680 ± 313.5@	9680 ± 248.32$
Neutrophil count/mm^3^	2774 ± 304.2	4100 ± 169.9*	2894 ± 189.6$	3921 ± 287.25#	3081 ± 267.34$
Lymphocyte count/mm^3^	4472 ± 384.8	9130 ± 235.2*	4872 ± 328.4$	6772 ± 343.2$	5892 ± 327.82$
Eosinophil count/mm^3^	184.8 ± 14.82	506.2 ± 36.71*	278 ± 19.67$	420.3 ± 19.45$	337.78 ± 13.26$
Monocyte count/mm^3^	109.3 ± 6.28	263.1 ± 21.52*	113.6 ± 8.67$	200.5 ± 17.08$	153.6 ± 8.31$

Values are expressed as mean ± SEM for six guinea pigs in each group

* *P* < 0.001 when compared with control group

@ *P* < 0.05

# *P* < 0.001

$ *P* < 0.001 when compared with sensitized group, BALF: Bronchoalveolar lavage fluid, TLC: Total leukocyte count

### Lung histology

**Figure 1:**
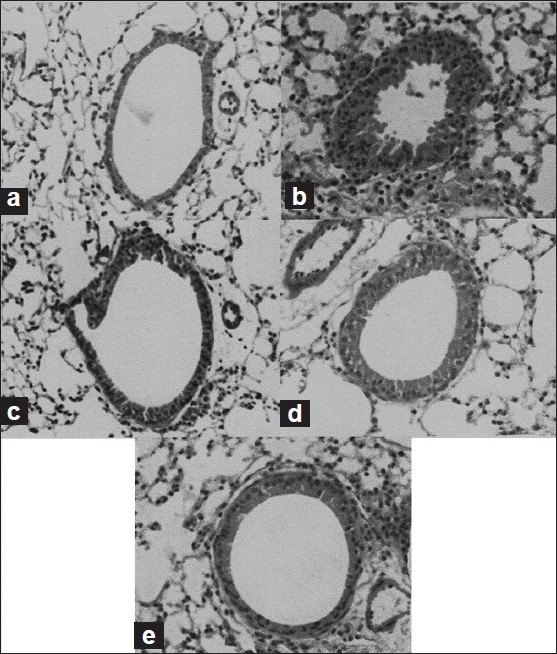
Effect of *Taxus baccata* on the histology of lung tissue. (a) Control group I, (b) egg albumin sensitized group II, (c) egg albumin sensitized + prednisolone (5 mg/kg) group III, (d) egg albumin sensitized + *T. baccata* treated (200 mg/kg, p.o.) group IV and (e) egg albumin sensitized + *T. baccata* treated (400 mg/kg, p.o.) group V. Prednisolone and *T. baccata* treatment was given for 15 days

Histological analysis of the lungs from non-sensitized, i.e., group I animals, showed normal lung histology [Figure 1a]. In contrast, similar to the BALF study, histological sections of lung tissue from group II guinea pigs exhibited airway inflammation, infiltration of eosinophils, lymphocytes and submucosal edema of the lungs, and bronchoconstriction shown as lumen plugging by mucus and cells [Figure 1b]. Treatment with prednisolone and *T. baccata*, i.e., group IV and group V animals, prevented the tissue edema, epithelial cell hypertrophy, infiltration of inflammatory cell, and airway lumen plugging, thereby decreasing inflammation and broncoconstriction, which led to normal lumen size [Figure 1c–e].

### Effect on compound 48/80 induced mast cell degranulation

AET and ketotifen were found to significantly (*P* < 0.001) inhibit rat peritoneal mast cell degranulation induced by compound 48/80 *in vitro* as compared to baseline value, i.e., +ve control group [Table 3].

**Table 3:** Effect of *Taxus baccata* on compound 48/80 induced mast cell degranulation

Treatment	Concentration (μg/ml)	Mast cells	
		% Granulated	% Degranulated
–ve control	–	91.83 ± 0.654	8.167 ± 0.654
+ve control	–	26.5 ± 1.176	73.5 ± 1.176
Ketotifen	10	80.5 ± 0.957*	19.5 ± 0.957
*Taxus baccata*	500	41.17 ± 0.945*	58.83 ± 0.945
*Taxus baccata*	750	50.67 ± 0.666*	49.33 ± 0.666
*Taxus baccata*	1000	63.17 ± 0.945*	36.83 ± 0.945

Values are expressed as mean ± SEM, *n* = 6 in each group

* *P* < 0.001 when compared with baseline value, i.e., +ve control

## DISCUSSION

Bronchial asthma is commonly characterized by increased airway reactivity to spasmogens. An initial event in asthma appears to be the release of inflammatory mediators like histamine, triggered by exposure to allergens that directly cause acute bronchoconstriction.[22,23] In the present study, histamine and acetylcholine were used as spasmogens in the form of aerosol to cause immediate bronchoconstriction in the form of PCD in guinea pigs. Bronchodilating effect of AET was evaluated by observing its effects at the time of PCD. In our study, we found that the time of occurrence of PCD was significantly increased, suggestive of bronchodilating activity following treatment with *T. baccata* against spasmogens.

Increasing evidence suggests that the frequently observed association between activated T lymphocytes and eosinophils plays a major role in the development of airway inflammation and in the accompanying bronchial hyperreactivity.[24,25] Neutrophils and monocytes play a pivotal role in the disease process as they are a source of variety of inflammatory mediators which are responsible for bronchial hyperresponsiveness and airway inflammation.[26] In association with asthma, elevated numbers of these inflammatory cells like eosinophils, neutrophils, lymphocytes, monocytes have been identified in various tissue compartments like blood, biopsies of lung tissue, in BALF and in sputum. In the present study, sensitization using egg albumin (1 ml, 10% w/v, i.p.) and then second exposure to the same antigen, i.e., egg albumin (0.5 ml, 2% w/v), through saphenous vein caused acute anaphylactic shock resembling the acute asthmatic attack resulting in the release of various mediators and cellular infiltration. Antigen challenge resulted in significant increase in the number of eosinophils in the BALF. This was accompanied by intense eosinophil infiltration, accumulation and degranulation in the guinea pig lungs as evident in histopathology study, which is consistent with human asthmatic lungs. In our study, we found that treatment with *T. baccata* in antigen challenged animals significantly inhibited antigen induced hyperreactivity by preventing increased infiltration of total leukocyte count and eosinophils count. After antigen challenge, airway hyperresponsiveness is supported by inflammatory pathology, suggesting the involvement of other mediators in the pathogenesis of asthma. Neutrophil numbers have also been reported to increase in bronchial lavage fluid in asthmatics, but neutrophilia is generally of shorter duration than eosinophilia.[27,28] This was observed in our result where treatment with *T. baccata* resulted in significant inhibition of antigen induced bronchial hyperreactivity by decreasing the neutrophil count. The participation of T lymphocytes in the pathogenesis of bronchial asthma and the accompanying bronchial hyperreactivity has been widely demonstrated.[25] Indeed, activated CD4^+^ T lymphocytes are found in the blood and bronchial lumen from asthmatics.[29] Recently, interest has been focused on the characterization of CD4^+^ T lymphocytes based on their repertoire of secreted cytokines and their possible role in the pathogenesis of allergic disorders. Thus, CD4^+^ T cells from asthmatics preferentially elaborate Th2-derived cytokines, such as IL-4 and IL-5, which have been shown to enhance IgE synthesis,[30] and to act specifically on eosinophil survival, activation, and secretion of proinflammatory mediators.[31] Large numbers of T lymphocytes, mainly of the CD4^+^ subset, have been identified in the bronchial mucosa of antigen challenged guinea pigs.[32] In accordance with the above, the present finding shows that treatment with *T. baccata* in the sensitized animals produced a significant decrease in the lymphocyte count as compared to the sensitized animals without treatment. The predominant cells in BALF recovered from unchallenged guinea pigs were those of the monocytes. The numbers of these cells were increased after antigen challenge.[33] In line with the above context, treatment with *T. baccata* significantly decreased monocytes as compared to that in sensitized guinea pigs. The results of our study suggest that in guinea pig airways, antigen challenge induced eosinophil, neutrophil, monocyte and lymphocyte infiltration and activation is similar to that reported in human asthmatics. These show that *T. baccata* exerts its protective effect by preventing the infiltration of inflammatory cell, thereby decreasing the release of preformed inflammatory mediators, which can prevent the direct damage to airway, which in turn prevents airway hyperresponsiveness.

Various processes involved in bronchial asthma such as inflammatory response can explain various histopathological alterations observed in the biopsy of asthmatic patients. In asthma, chronic inflammation is responsible for the bronchoconstriction which leads to airway narrowing and decrease in the lumen size of the bronchiole.[34] This can be clearly seen by observing the cross-section of bronchi in the histopathological studies of the lung tissue. In the present study, the sections of the lung tissues of animals sensitized with egg albumin depicted marked bronchitis and severe bronchoconstriction. Treatment with *T. baccata* prevented the inflammation and bronchoconstriction, which led to normal lumen size and normal cellular structure, compared to antigen-sensitized guinea pigs.

Mast cell degranulation is important in the initiation of immediate responses following exposure to allergens.[35] Once binding of allergen to cell-bound IgE occurs, mediators such as histamine; eosinophil and neutrophil chemotactic factors; leukotrienes C4, D4, and E4; prostaglandins; platelet-activating factor; and others are released from mast cells, which are responsible for the development of airway inflammation and bronchoconstriction. An attempt was made to find out whether AET has any effect on the rate of disruption of mast cells following exposure to compound 48/80, an agent which causes histamine release.[36] It has been assumed that the process leading to histamine secretion may be mediated by calcium release from an intracellular store of mast cells.[37] In this study, *T. baccata* offered significant protection against compound 48/80 induced mast cell degranulation by stabilizing it, which is responsible for the decreasing airway inflammation by preventing the release of various inflammatory mediators.

Phytochemical screening of *T. baccata* showed the presence of lignans, flavonoids, sugar derivatives, etc.[38,39] Lignans are known to possess various biological activities including antibacterial, antioxidant, anticancer, spasmolytic and anti-inflammatory effects.[40] Flavonoids are known to possess various biological activities including antibacterial, antifungal, spasmolytic, antiviral, anticancer, and anti-inflammatory effects.[41–43] Anti-asthmatic activity of *T. baccata* may be due to the presence of the above constituents. In conclusion, our data suggest that the alcoholic extract of the leaves of *T. baccata* possesses significant anti-asthmatic activity and has beneficial effect in asthma by causing bronchorelaxation and decreasing bronchial hyperreactivity.
